# Two new *Inosperma* (Inocybaceae) species from the karst forest of Guangxi, China

**DOI:** 10.3897/mycokeys.132.187183

**Published:** 2026-05-26

**Authors:** Yan Cheng Zhang, Shuang Li, Yu-Guang Fan, Jin Rong Liu, Wei Ning Tan, Yan Liu, Guang Fu Mou

**Affiliations:** 1 Nonggang Karst Ecosystem Observation and Research Station of Guangxi, Guangxi Institute of Botany, Guangxi Zhuang Autonomous Region and Chinese Academy of Sciences, Guilin, 541006, Guangxi, China Engineering Research Center of Tropical Medicine Innovation and Transformation of Ministry of Education, Hainan Provincial Key Laboratory of Research and Development on Tropical Herbs, School of Pharmacy, Hainan Medical University Haikou China; 2 Engineering Research Center of Tropical Medicine Innovation and Transformation of Ministry of Education, Hainan Provincial Key Laboratory of Research and Development on Tropical Herbs, School of Pharmacy, Hainan Medical University, Haikou, China Nonggang Karst Ecosystem Observation and Research Station of Guangxi, Guangxi Institute of Botany, Guangxi Zhuang Autonomous Region and Chinese Academy of Sciences Guilin China; 3 Administrative Centre of Guangxi Mulun National Nature Reserve, Huanjiang 547100, Guangxi, China Administrative Centre of Guangxi Mulun National Nature Reserve Huanjiang China

**Keywords:** Agaricales, new taxa, phylogeny, taxonomy

## Abstract

In this study, we described two new species, *Inosperma nonggangense* Y.G. Fan, G.F. Mou & S. Li, **sp. nov**. and *Inosperma rufobrunneum* Y.G. Fan, G.F. Mou & S. Li, **sp. nov**. from Guangxi, China. These species were identified based on morphological and multi-locus (ITS, LSU, and *rpb*2) phylogenetic analyses. *Inosperma nonggangense***sp. nov**. is characterized by yellowish to orange basidiomata, rimulose pileus, crowded lamellae, ovoid to ellipsoid basidiospores measuring 8.7–9.9 × 5.2–6.1 μm, thin-walled, broadly clavate cheilocystidia, and abundant oily hyphae in hymenophoral, pileal, and stipe trama. *Inosperma rufobrunneum***sp. nov**. is characterized by reddish-brown basidiomata at maturity, rimulose to rimose pileus, crowded lamellae, nearly smooth stipes, ellipsoid basidiospores measuring 8.2–9.3 × 4.9–5.4 μm, and thin-walled, clavate cheilocystidia. Phylogenetically, these species belong to the Old World tropical clade 2, formed distinct independent lineages.

## Introduction

*Inosperma* was initially established as a section of *Inocybe* s.l., with *Inoc. calamistrata* Kühner as its type species (Kühner 1980). Matheny et al. (2020) elevated it to one of the seven genera within the family Inocybaceae. Members of *Inosperma* generally have small to medium fruiting bodies, caps with cracks or scales, smooth or scaly stipes with bases that are orderly or bulbous, sometimes showing bruising color reactions, smooth, oval to bean-shaped or nearly spherical spores, transparent or necro-pigmented basidia, thin-walled cheilocystidia, lack of pleurocystidia, and occasionally with special odors such as earthy, fishy, aromatic, fruity, geranium, truffle, etc. (Kuyper 1986; Matheny et al. 2020; Deng et al. 2021a, 2021b).

In recent years, numerous new species continue to be described from all over the world (Deng et al. 2021a, 2021b; Deng et al. 2022; Li et al. 2022; Aïgnon et al. 2023; Zhou et al. 2023; Crous et al. 2024; Sana et al. 2025). The number of *Inosperma* names registered in the Index Fungorum database has increased to 99 (as of February, 2026), and the genus is distributed on all continents except South America (Matheny et al. 2020). Multigene (ITS, nrLSU, and *rpb*2) molecular studies have confirmed that *Inosperma* is monophyletic and has been divided into six major lineages, namely the *Inos. africanum* lineage, the section *Cervicolores*, the *Inos. misakaense* lineage, the *Maculatum* clade, Old World tropical clade 1, and Old World tropical clade 2 (Matheny et al. 2020; Deng et al. 2021a). Among these clades, taxa from tropical Asia (the Old World tropical clade 2) have typically been responsible for numerous poisoning incidents in India (Chandrasekharan et al. 2020), Thailand (Parnmen et al. 2021), and tropical China (Deng et al. 2022). Accordingly, clarifying species diversity and their distribution is crucial for preventing and treating poisoning cases.

Guangxi Zhuang Autonomous Region (Abbreviated as Guangxi) is located at the southeastern edge of the Yunnan-Guizhou Plateau and in the western part of the Guangdong-Guangxi Hills. It serves as a transition zone between the second and third steps of China’s terrain and, more importantly, as a vital ecological barrier in southern China. Guangxi, stretching from south to north, spans three climatic zones and encompasses four priority areas for biodiversity conservation in China. It is thus recognized as one of the provinces with the richest biodiversity in China. Guangxi also lies within the core area of China’s distinctive southwestern karst landform, being the province with the highest concentration and most extensive development of karst landforms (Tan et al. 2023). The complex and diverse karst habitats serve as ideal habitats for numerous endemic species and as breeding grounds for many new taxonomic groups. To date, 9,051 plant species belonging to 2,073 genera and 307 families have been recorded in Guangxi (Chen & Huang, 2025). The region’s great variety of vegetation types has fostered abundant macrofungal resources. Available data indicate that 1,145 macrofungal species have been recorded in Guangxi, comprising 163 Ascomycota and 982 Basidiomycota (Wu et al. 2021), and many new fungal species have been described from Guangxi (Bao et al. 2025; Liu et al. 2025). Furthermore, regions with greater plant diversity typically exhibit a higher potential for the diversification of symbiotic fungi. As an Agaricales fungus, *Inosperma* establishes an ectomycorrhizal symbiosis with trees, and the diverse vegetation in Guangxi likely plays a key role in driving the diversification of this genus.

Here, we describe two new species from southwestern China, namely *Inos. nonggangense* and *Inos. rufobrunneum*, based on morphological characteristics and multigene molecular analyses using combined ITS, LSU, and *rpb*2 sequence data. This study provides comprehensive macroscopic and microscopic descriptions, color photographs, illustrations, and discussions of these species.

## Materials and methods

### Specimen collection

Specimens used in the present work were collected from the tropical and subtropical karst forest in Guangxi Zhuang Autonomous Region, China. Fresh basidiomata were photographed with a digital camera, and macroscopic characteristics and other relevant collection data (Rathnayaka et al. 2025) were recorded as field notes. The specimens were dried in a portable electric oven at 45 °C overnight. Dried specimens were deposited in Herbarium of Guangxi Institute of Botany (IBK, Guangxi, China).

### Morphological study

Macro-morphological descriptions were based on field records and measurements of the specimens in the herbarium. Micro-morphological data were obtained from dried specimens and observed under an optical microscope. Color codes followed the standards established by Kornerup et al. (1978). Microscopic features were studied on dried samples after hand sectioning, rehydration in distilled water, and, if necessary, mounting in Congo red. The study utilized a Nikon E80i microscope to examine the sections.

The basidiospores dimension is described as follows: [*a*/*b*/*c*] length × width, and the factor Q. Factor Q is the ratio of spore length to width. The measured value from *a* basidiospore of *b* basidiomata in *c* specimens is shown by (*d*) *e*–*f* (*g*), where *e*–*f* represents at least 90% range of the values, *d* the minimum value, and *g* the maximum value. One hundred mature matured basidiospores were measured for each new species.

### DNA extraction, PCR amplification, and sequencing

DNA from basidiomata was extracted using an EZup Fungi Genomic DNA Purification Kit (Sangon Biotech, Shanghai, China). PCR amplifications were conducted using the following primers: ITS4 and ITS5 for the internal transcribed spacer region (ITS) (Gardes and Bruns 1993), LR0R and LR7 for the large subunit nuclear ribosomal RNA (LSU) (Vilgalys and Hester 1990), and bRPB2-6F and bRPB2-7.1R for second largest subunit of RNA polymerase II (*rpb*2) (Matheny 2005). Polymerase chain reaction (PCR) was performed in a total volume of 30 μL, consisting of 15 μL of 2× Es Taq MasterMix, 9 μL of ddH_2_O, 1.5 μL of each upstream and downstream primer, and 3 μL of DNA template. The PCR procedure for ITS, LSU and *rpb*2 were as follows: initial denaturation at 95 °C for 1 min, followed by 35 cycles of denaturation at 95 °C for 30 s, annealing at 52 °C for 1 min, and extension at 72 °C for 1 min, with a final extension at 72 °C for 8 min. The resulting PCR products were sent to Sangon Biotech for sequencing.

### Phylogenetic analyses

The generated sequences were checked for their quality, assembled and subjected to BLAST searching (https://blast.ncbi.nlm.nih.gov/Blast.cgi), and sequences in the database with more than 90% homology were downloaded. In addition, two *Auritella* (TH10009 and TH9866) sequences were retrieved and included as outgroups. Sequences were aligned using E-INS-i iterative refinement methods on the MAFFT online service (https://www.ebi.ac.uk/Tools/msa/mafft/) and processed using BioEdit v7.0.9.0 (Hall 1999). The sequences were subsequently concatenated together in MEGA7 (Kumar et al. 2016) and subjected to Maximum Likelihood (ML) and Bayesian inference (BI) analyses. The ML analysis was conducted with the IQ-TREE web server (http://iqtree.cibiv.univie.ac.at/) using the ultrafast bootstrap analysis option and SH-aLRT branch testing, with bootstrap alignments set to 1,000, maximum iterations set to 1,000, and the minimum correlation set to 0.99 (Trifinopoulos et al. 2016). For the BI analysis, the best-fit evolutionary model for each locus was first estimated using MrModeltest v2.3 (Nylander 2004). The BI analysis was carried out in MrBayes 3.2.7 and consisted of 4 chains of 1,000,000 generations, each sampled every 100 generations. The analysis was halted when the average standard deviation of split frequencies fell below 0.01; the first 25% of trees were discarded, and the results were aggregated using the “sump” and “sumt” commands (Ronquist et al. 2012). Finally, the phylogenetic tree was visualized in FigTree v1.4.0 and modified and refined using the TVBOT online tool (https://www.chiplot.online/tvbot.html).

## Results

### Phylogenetic inference

As shown in Table 1, a total of 324 sequences (122 of ITS, 115 of LSU, and 87 of *rpb*2), including 17 new sequences (eight of ITS, seven of LSU, and two of *rpb*2) that were submitted to GenBank, were analyzed in this study. The final alignment comprised 2,509 nucleotides (733 of ITS, 1,103 of LSU, and 673 of *rpb*2) including gaps, and was deposited in TreeBASE (ID32453). For the BI analysis, the best-fit substitution models selected for each data partition using MrModeltest were GTR+I+G equally. The BI phylogenetic analysis was stopped after 7555,000 MCMC generations, when the average standard deviation of split frequencies converged to 0.009702, the average effective sample size was 4662.97, and the average potential scale reduction factor parameter value was 1.001. The following models, automatically selected by IQ-TREE, were used for the ML analysis, yielding a final log-likelihood value of −34735.38: SYM+I+G for ITS, GTR+I+G for 28S, and GTR+I+G for *rpb*2.

**Table 1.** Taxa, sequences, and collections analyzed in this study. Collections in bold are newly sequenced in this study. The Type after the voucher indicate the specimens is from holotype. ―indicates sequences that are not available.

Species	Voucher	Country	GenBank accession			References
			ITS	LSU	*rpb*2	
*Auritella hispida*	TH10009	Cameroon	KT378203	KT378207	KT378215	Matheny et al. (2017)
*A. spiculosa*	TH9866	Cameroon	KT378204	KT378206	KT378214	Matheny et al. (2017)
*Inosperma acutofulvu*m	MCVE29416	Italy	MG944832	―	―	Bizio and Castellan (2017)
*Inos. adaequatum*	JV16501F	Finland	―	AY380364	AY333771	Matheny et al. (2020)
*Inos.* aff. lanatodiscum	PBM3051	USA	JQ801401	JN975026	JQ846485	Pradeep et al. (2016)
*Inos.* aff. calamistratum	DED8134	Thailand	GQ892983	GQ892937	―	Pradeep et al. (2016)
*Inos.* aff. calamistratum	REH8420	Costa Rica	JQ801390	JN975018	JQ846471	Esteve-Raventós et al. (2024)
*Inos.* aff. *fastigiellum*	PBM3325	USA	JQ801399	JQ815419	JQ846477	Pradeep et al. (2016)
*Inos.* aff. latericium	TR109-02	PNG	JQ801405	JN975023	JQ846487	Pradeep et al. (2016)
*Inos. africanum*	HLA0383 (Type)	Benin	MT534298	MT560733	―	Aïgnon et al. (2021)
*Inos. africanum*	HLA0353	Benin	MT534299	―	―	Aïgnon et al. (2021)
*Inos. afromelliolens*	PC96013	Zambia	JQ801383	JQ815408	EU600882	Matheny and Moreau (2009a)
*Inos. akirnum*	CAL1358	India	KY440085	KY549115	KY553236	Latha and Manimohan (2017)
*Inos. apiosmotum*	PBM3020	USA	JQ801385	JN975021	JQ846463	Unpublished
*Inos. bicoloratum*	ZT12187	Malaysia	GQ892984	GQ892938	JQ846464	Horak et al. (2015)
*Inos. bongardii*	JV7450F	Finland	―	EU555448	―	Matheny et al. (2009b)
*Inos. bulbomarginatum*	MR00357	Benin	MN096190	MN200775	MN097882	Aïgnon et al. (2021)
*Inos. bulbomarginatum*	PC96082	Benin	JQ801412	JN975027	―	Aïgnon et al. (2021)
*Inos. calamistratoides*	PBM3384	Australia	JQ801393	JQ815415	KJ729949	Kropp et al. (2013)
*Inos. calamistratum*	PBM1105	USA	JQ801386	JQ815409	JQ846466	Esteve-Raventós et al. (2024)
*Inos. calamistratum*	EL1904	Sweden	AM882938	AM882938	―	Esteve-Raventós et al. (2024)
*Inos. calamistratum*	PBM2351	USA	―	AY380368	AY333764	Esteve-Raventós et al. (2024)
*Inos. calamistratum*	JV11950	Latvia	―	EU555452	AY333763	Esteve-Raventós et al. (2024)
*Inos. calamistratum*	TR74-06	PNG	JQ801391	JN975020	JQ846472	Esteve-Raventós et al. (2024)
*Inos. carnosibulbosum*	TBGT12047	India	KT329448	KT329443	KT329454	Pradeep et al. (2016)
*Inos. cervicolor*	TURA4761	Finland	JQ801395	JQ815417	JQ846474	Pradeep et al. (2016)
*Inos.* cf. gregarium	D82	Thailand	MW538596	MW512894	MW538609	Parnmen et al. (2021)
*Inos.* cf. gregarium	D84	Thailand	MW538598	MW512895	MW538610	Parnmen et al. (2021)
*Inos.* cf. gregarium	D91	Thailand	MW538602	MW512896	MW538611	Parnmen et al. (2021)
*Inos.* cf. gregarium	D92	Thailand	MW538603	MW512897	MW538612	Parnmen et al. (2021)
*Inos.* cf. lanatodiscum	TURA1812	Finland	JQ408763	JQ319694	JQ846484	Pradeep et al. (2016)
*Inos.* cf. *reisneri*	MCA646	Japan	―	EU555463	―	Pradeep et al. (2016)
*Inos. changbaiense*	FYG2010156 (Type)	China	MH047251	MG844976	MT086755	Bau and Fan (2018)
*Inos. cyanotrichium*	I37	Australia	JQ801396	JN975033	JQ846476	Pradeep et al. (2016)
*Inos. dodonae*	SMNS-STU-F-0901253 (STU)	Netherlands	MW647615	―	―	Bandini et al. (2021)
*Inos. erubescens*	JV9070F	Finland	―	EU569846	―	Pradeep et al. (2016)
*Inos. flavobrunneum*	HLA0367	Benin	MN096199	MT536754	―	Aïgnon et al. (2021)
*Inos. geraniodorum*	EL10606	Sweden	FN550945	FN550945	―	Esteve-Raventós et al. (2024)
*Inos. gregarium*	CAL1309	India	KX852305	KX852307	KX852306	Latha and Manimohan (2016)
*Inos. gregarium*	ZT8944	India	―	EU600903	EU600902	Pradeep et al. (2016)
*Inos. hainanense*	Zeng4737	China	MZ373980	―	MZ388091	Deng et al. (2021b)
*Inos. ismeneanum*	SMNS-STU-F-0901561 (STU)	Germany	MW647625	―	―	Bandini et al. (2021)
*Inos. lanatodiscum*	PBM2451	USA	JQ408759	JQ319690	JQ846483	Pradeep et al. (2016)
*Inos. latericium*	PDD92382	New Zealand	GU233367	GU233413	―	Pradeep et al. (2016)
*Inos. longisporum*	MHNNU32337 (Type)	China	OP135509	OP135495	OP161560	Li et al. (2022)
*Inos. misakaense*	PC96234	Zambia	JQ801409	EU569875	AY333767	Pradeep et al. (2016)
*Inos. monastichum*	STU: SMNS-STU-F-0901533	Germany	MW647631	―	―	Bandini et al. (2021)
*Inos. mucidiolens*	DG1824 (Type)	Canada	HQ201339	HQ201340	―	Pradeep et al. (2016)
*Inos. muscarium*	FYG6091 (Type)	China	MZ373982	MZ388093	MZ373991	Deng et al. (2021b)
*Inos. mutatum*	PBM2542	USA	―	AY732212	DQ472729	Matheny et al. (2006)
*Inos. neobrunnescens*	PBM2452	USA	―	EU569868	EU569867	Horak et al. (2015)
*Inos. neobrunnescens* var. *leucothelotum*	SAT0427406	USA	JQ801411	JN975025	JQ846489	Pradeep et al. (2016)
*Inos. proximum*	ZT13015	Thailand	EU600839	EU600840	―	Matheny et al. (2020)
*Inos. quietiodor*	EL11504	Sweden	AM882960	AM882960	―	Pradeep et al. (2016)
*Inos. rhodiolum*	EL223-06	France	FJ904175	FJ904175	―	Pradeep et al. (2016)
*Inos. rimosoides*	PBM2459	USA	DQ404391	AY702014	DQ385884	Pradeep et al. (2016)
*Inos. rubricosum*	PBM3784	Australia	KP308817	KP170990	KM406230	Pradeep et al. (2016)
*Inos. saragum*	CAL1360	India	KY440103	KY553249	KY549133	Latha et al. (2017)
*Inos. shawarense*	ASSE79	Pakistan	KY616964	KY616966	―	Naseer (2018)
*Inos.* sp.	L-GN3a	PNG	JX316732	―	JX316732	Pradeep et al. (2016)
*Inos.* sp.	TJB10045	Thailand	KT600658	KT600660	KT600659	Pradeep et al. (2016)
*Inos.* sp.	TR220-06	PNG	JQ801416	JQ846496	JN975017	Pradeep et al. (2016)
*Inos.* sp.	D67	Thailand	MW538582	MW512898	MW538613	Unpublished
*Inos.* sp.	D68	Thailand	MW538583	MW512899	MW538614	Unpublished
*Inos.* sp.	D76	Thailand	MW538590	MW512900	MW538615	Unpublished
*Inos.* sp.	D77	Thailand	MW538591	MW512901	MW538616	Unpublished
*Inos.* sp.	D78	Thailand	MW538592	MW512902	MW538617	Parnmen et al. (2021)
*Inos.* sp.	D80	Thailand	MW538594	MW512903	MW538618	Parnmen et al. (2021)
*Inos.* sp.	D83	Thailand	MW538597	MW512904	MW538619	Parnmen et al. (2021)
*Inos.* sp.	D85	Thailand	MW538599	MW512905	MW538620	Parnmen et al. (2021)
*Inos.*sp.	D86	Thailand	MW538600	MW512906	MW538621	Parnmen et al. (2021)
*Inos.*sp.	D89	Thailand	MW538601	MW512907	MW538622	Parnmen et al. (2021)
*Inos.*sp.	D152	Thailand	MW538604	MW512908	MW538623	Parnmen et al. (2021)
*Inos.* sp.	D324	Thailand	MW538607	MW512909	MW538624	Unpublished
*Inos.*sp.	PBM2871	USA	HQ201348	HQ201348	JQ846475	Unpublished
*Inos.* sp.	BB3233	Zambia	JQ801415	EU600885	―	Pradeep et al. (2016)
*Inos. sphaerobulbosum*	MHHNU32266 (Type)	China	OP135501	OP134001	OP161559	Li et al. (2022)
*Inos. squamulosobrunneum*	MHHNU32359 (Type)	China	OP135499	OP134000	OP161562	Li et al. (2022)
*Inos. squamulosohinnuleum*	MHHNU32195	China	OP135500	OP134002	OP161558	Li et al. (2022)
*Inos. subhirsutum*	PC96073	Zambia	JQ801417	EU600870	EU600869	Esteve-Raventós et al. (2024)
*Inos. subsphaerosporum*	FYG5848 (Type)	China	MW403825	MW404237	MW397171	Deng et al. (2021a)
*Inos. vinaceobrunneum*	PBM2951	USA	―	HQ201353	JQ846478	Pradeep et al. (2016)
*Inos. vinaceum*	AMB18747	Italy	MW561108	MW561120	―	Cervini et al. (2020)
*Inos. viridipes*	I153	Australia	KP641646	KP171095	KM656139	Unpublished
*Inos. virosum*	TBGT753	India	KT329452	KT329446	KT329458	Pradeep et al. (2016)
*Inos. virosum*	CAL1383	India	KY440108	KY553253	KY549138	Latha and Manimohan (2017)
*Inos. wuzhishanense*	FYG7659 (Type)	China	OR436481	OR436487	―	Zhou et al. (2023)
*Inos. wuzhishanense*	FYG7343	China	OR436482	OR436488	OR451201	Zhou et al. (2023)
*Inos. zonativeliferum*	FYG6441 (Type)	China	OL850878	OM845772	ON075044	Deng et al. (2021b)
*Inos. africanum*	MR00387	Benin	MN096189	MN097881	MT770739	Aïgnon et al. (2021)
*Inos. afromelliolens*	HLA0754	Benin	OQ300372	OQ300369	OQ435246	Aïgnon et al. (2023)
*Inos. afromelliolens*	OF4026	Benin	PQ552962	PQ528055	―	Unpublished
*Inos. afromelliolens*	PC96080 (Type)	Zambia	OQ441164	OQ300372	OQ435246	Matheny et al. (2009b)
*Inos. apollonium*	SMNS-STU-F-0901670 (Type)	Austria	NR_184499	ON003428	―	Bandini et al. (2022)
*Inos. calamistratum*	AH40200	Spain	PP431518	PP431538	PP478162	Esteve-Raventós et al. (2024)
*Inos. calamistratum*	AH46636	Portugal	PP431514	PP431535	PP478161	Esteve-Raventós et al. (2024)
*Inos. calamistratum*	EL36219	France: Corsica	OR803784	OR803784	―	Esteve-Raventós et al. (2024)
*Inos. geminum*	EL6306	Norway	OR823936	OR823936	PP092172	Esteve-Raventós et al. (2024)
*Inos. geminum*	JV 31497 (TUR, Type)	Sweden	OR823936	OR823936	PP092174	Esteve-Raventós et al. (2024)
*Inos. geraniodorum*	J. Favre Z.A.82b (G00052203, Type)	Switzerland	PP431545	―	―	Esteve-Raventós et al. (2024)
*Inos. geraniodorum*	EL15617	Sweden	OR823942	OR823942	PP092176	Esteve-Raventós et al. (2024)
*Inos. gracilentum*	EL 85-19 (GB 0207620, Type)	Sweden	OR817726	OR817726	―	Esteve-Raventós et al. (2024)
*Inos. gracilentum*	ZA82a	Switzerland: Grisons	PP431548	―	―	Esteve-Raventós et al. (2024)
*Inos. macrocarpum*	HLA0787	Africa	OQ300390	OQ286290	OQ427873	Aïgnon et al. (2023)
*Inos. macrocarpum*	HLA0788	Africa	OQ300391	OQ286291	OQ435242	Aïgnon et al. (2023)
*Inos. macrocarpum*	HLA0790	Africa	OQ300392	OQ286292	OQ435243	Aïgnon et al. (2023)
*Inos. macrocarpum*	HLA0791 (Type)	Africa	OQ300373	OQ300370	OQ435244	Aïgnon et al. (2023)
*Inos. macrocarpum*	HLA0792	Africa	OQ300373	OQ300370	OQ435244	Aïgnon et al. (2023)
*Inos. monastichum*	SMNS-STU-F-0901533 (STU, Type)	Germany	MW647631	―	―	Bandini et al. (2021)
***Inos. nonggangense***	**M2018102624**	**China**	**PX761791**	―	―	**This study**
***Inos. nonggangense***	**M2018061321** (Type)	**China**	**PX643276**	**PX629743**	―	
***Inos. nonggangense***	**M2018092505**	**China**	**PX643277**	**PX629744**	―	
*Inos. kashmiranum*	N277, LAH38340 (Type)	Pakistan	PQ449774	PQ449776	PQ584605	Naseer et al. (2025)
*Inos. praetermissum*	AH46901 (Type)	Andorra	PP431546 (ITS1) / PP431550 (ITS2)	―	―	Esteve-Raventós et al. (2024)
*Inos. praetermissum*	EL7019	Sweden	OR831123	―	―	Esteve-Raventós et al. (2024)
***Inos. rufobrunneum***	**DMS2022071619**	**China**	**PX629738**	**PX629733**	**―**	**This study**
***Inos. rufobrunneum***	**M2018092217**	**China**	**PX629739**	**PX629734**	**―**	
***Inos. rufobrunneum***	**M2020072503** (Type)	**China**	**PX629740**	**PX629735**	**PX508876**	
***Inos. rufobrunneum***	**M2020072512**	**China**	**PX629741**	**PX629736**	**―**	
***Inos. rufobrunneum***	**M2020072531**	**China**	**PX629742**	**PX629737**	**PX508877**	
*Inos. subhirsutum*	EB 1992082601 (dupl. AH 56195)	Italy	PP431507	PP431530	―	Esteve-Raventós et al. (2024)
*Inos. turietoense*	AH47710 (Type)	Spain	PP431526	PP431541	PP478208	Esteve-Raventós et al. (2024)
*Inos. veliferum*	R. Kühner71-143 (G00110853, Type)	France	PP431520	―	―	Esteve-Raventós et al. (2024)
*Inos. adaequatum*	0229744 (Type)	―	KM873367	JQ815407	AY333771	Marchetti et al. (2014)
*Inos. cyanotrichium*	TENN065729	Australia	JQ801397	KJ729948	―	Unpublished
*Inos. viridipes*	TENN066999 (Type)	Australia	NR_153168	―	―	Pradeep et al. (2016)
*Inos. pavithrum*	DKP-SERB65	India	PP350421	PP192110	PP209642	Crous et al. (2024)
*Inos. subsphaerosporum*	FYG6177	China	OQ694986	OQ690103	OQ708947	Deng et al. (2021a)
*Inos. maculatum*	PBM2446	Sweden	―	―	EU569863	Pradeep et al. (2016)
*Inos. calamistratoides*	ZT9630	New Zealand	JQ801392	JQ815413	―	Pradeep et al. (2016)
*Inos. neohirsutum*	AH 26947 (Type)	Spain	PP431510	PP431532		Esteve-Raventós et al. (2024)
*Inos. apiosmotum*	TENN062582	USA	JQ801384	―	―	Unpublished
*Inos. apiosmotum*	F043329	Canada	NR_121487	―	―	Unpublished

**Figure 1.**
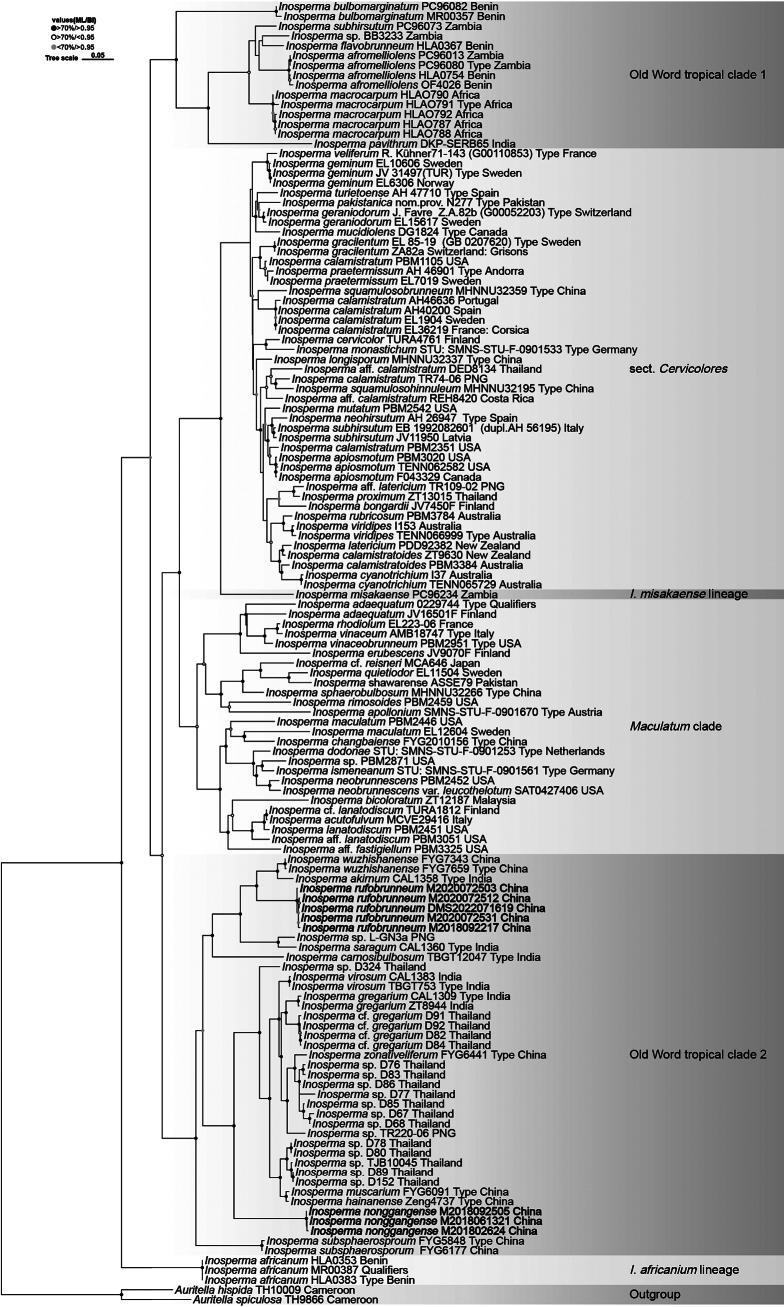
Maximum likelihood phylogenetic tree of a combined dataset of *Inosperma* species sequences (ITS, LSU, and *rpb*2), with *Auritella spiculosa* (TH9866, Cameroon) and *A. hispida* (TH10009, Cameroon) used as outgroups. Black circle at the nodes indicate > 70% / > 0.95, hollow circle indicate > 70% / < 0.95, and gray circle indicate < 70% / > 0.95, following the format (ML-BP/BI-PP) *Inosperma rufobrunneum* and *Inos. nonggangense* are two newly described taxa.

In the generated phylogenetic trees, each of the new species was clearly distinct, with all specimens of a given species clustering together as expected. Although the phylogenetic trees constructed by ML and BI differed slightly, their topologies were basically the same. The ML tree is therefore shown in Fig. 1, with only those nodes with BI posterior probabilities exceeding 0.95 and ML bootstrap support values above 70% were included.

As is shown in Fig. 1, phylogenetic analyses strongly supported the placement of the two new taxa within the family Inocybaceae and the genus *Inosperma*, both of which belong to the Old World Tropical Clade 2. *Inosperma wuzhishanense* Y.G. Fan, L.S. Deng & W.J. Yu, and *Inos. akirnum* (K.P.D. Latha & Manim.) Matheny & Esteve-Rav. formed a sister group, and the distinct lineage composed of these two taxa was most closely related to *Inos. rufobrunneum* sp. nov., which included five specimens. Meanwhile, the specimens M2018092505, M2018061321, and M201802624 formed a relatively independent branch at the base of the Old World Tropical Clade 2, and this branch was identified as *Inos. nonggangense* sp. nov.

### Taxonomy

#### Inosperma rufobrunneum

Taxon classificationFungiAgaricalesInocybaceae

Y.G. Fan, G.F. Mou & S. Li sp. nov.

C25DE1EB-9BDE-5F3E-8136-407FCF213609

Fungal Names: FN 573254

Figs 2, 3

##### Chinese name.

红褐歧盖伞 (Pinyin: hóng hè qí gài sǎn).

**Figures 2.**
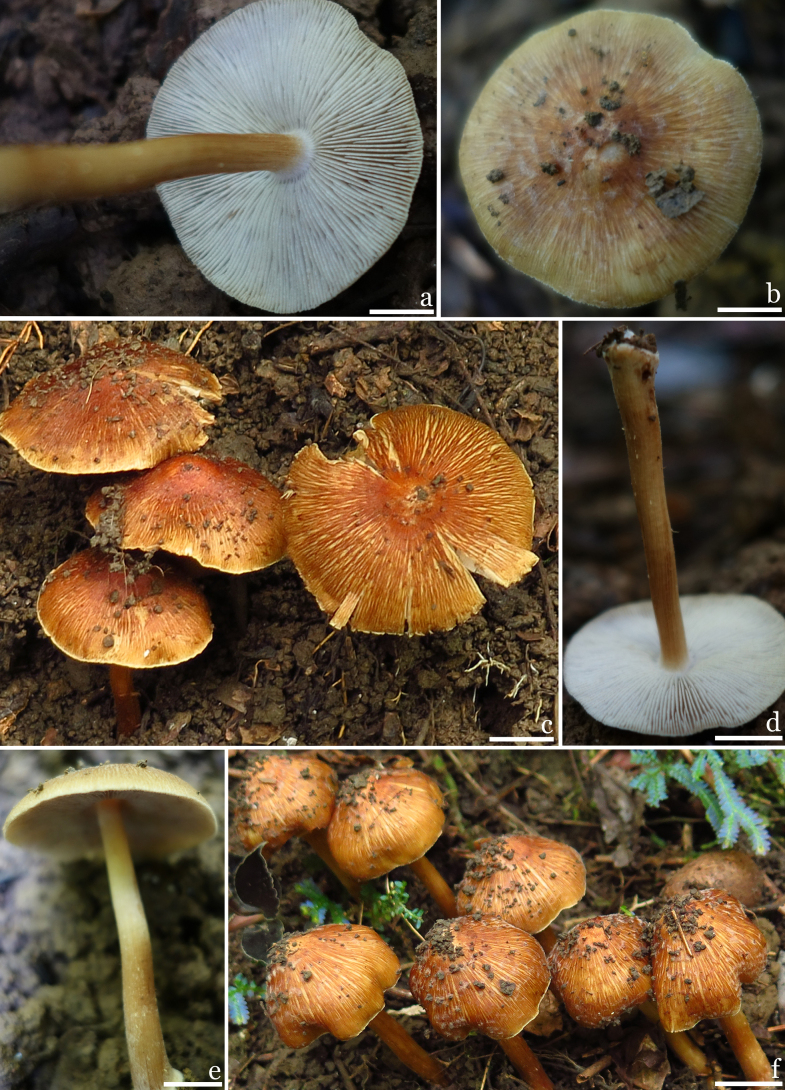
Basidiomata of *Inosperma rufobrunneum*. (M2020072503, holotype). **a**. Lamellae; **b–c**. Pileus; **d–e**. Stipe surface; **f**. Basidiomata. Scale bars: 10 mm (**a–f**). Photos by G.F. Mou.

**Figures 3.**
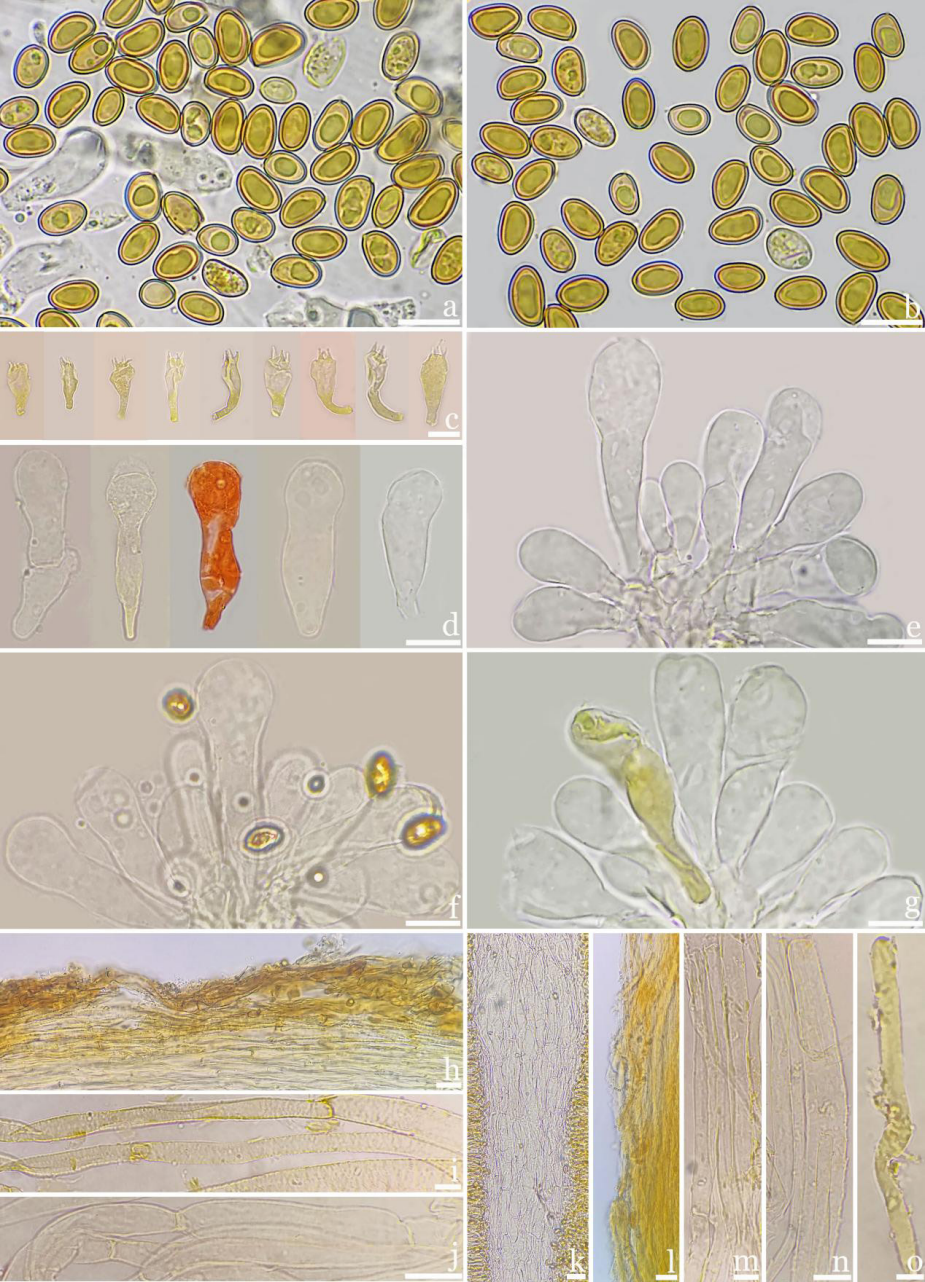
Microscopic features of *Inosperma rufobrunneum* (M2020072503, holotype). **a–b**. Basidiospores; **c**. Basidia; **d–g**. Cheilocystidia; **h**. Pileipellis; **i**. Hyphae of pileipellis; **j**. Hyphae of pileal trama; **k**. Hymenophoral trama; **l**. Stipitipellis; **m**. Stipe hypha; **n**. Stipe trama; **o**. Oleiferous hyphae. Scale bars: 10 μm (**a–o**).

##### Etymology.

*Rufobrunneum* (Lat.): refers to the color of the pileus at maturity, which ranges from pale yellowish-brown to reddish-brown.

##### Holotype.

**China** • Guangxi Zhuang Autonomous Region, Hechi City, Huanjiang Maonan Autonomous County, Mulun National Nature Reserve, occurring in fagaceous forests, 25°06'50"N, 107°57'53"E, 25 July 2020, G.F. Mou, M2020072503 (IBK), GenBank accession number: (ITS: PX629740; LSU: PX629735 and *rpb2*: PX508876).

##### Diagnosis.

*Inosperma rufobrunneum* has reddish-brown basidiomata when mature, crowded lamellae, ellipsoid basidiospores, and thin-walled non-hyaline cheilocystidia with a tapered base. Most similar to *Inos. wuzhishanense*, but differs from it by the non-vinaceous tinged basidiomata, firbrillose-rimulsoe pileus, elongate-ellipsoid spores, and clavate cheilocystidia that are distinctly tapered downwards.

##### Description.

***Basidiomata*** medium-sized. ***Pileus*** 24–40 mm in diameter, conical when young, becoming campanulate to plano-campanulate upon maturity, not fully expanding; margin incurved when immature, then bends downward, and finally spreading when mature; surface dry, rough, covered with appressed reddish-brown (6C8) fine fibrils, and initially enveloped in white (1A1) to off-white (1A1–1B1) veil remnants, appearing fibrillose-rimulose to finely cracked, sometimes distinctly fissured; yellowish brown (4A3), reddish-brown (6B8) when young, reddish-brown (6B8) to reddish-brown (6C8) when mature, dark in the middle, fading toward the margin. ***Lamellae*** rather crowded, adnate, 2–3 mm wide, unequal length, distribute 3–4 small folds, with indistinct fimbriate edges; initially white (1A1), gradually turning grayish-white (1A1–1B1) to brown (6C6), and dark brown (5E7) at maturity. ***Stipe*** 44–65 × 4–6 mm, central, solid, rough, cylindrical or slightly curved, with a sub-bulbous base that swells to 10 mm wide; exhibiting longitudinal striate-fibrillose patterns throughout, formed by dense reddish-brown (6C8) fine fibrils; pale yellowish-brown (4A3) to reddish-brown (6B8), reddish-brown (6C8) when mature, with a light yellowish-brown top (4A2) and a white (1A1) to reddish-brown (6C7) base. ***Context in pileus*** fleshy, white (1A1), about 2–3 mm thick, and 3 mm thick in the center of the pileus, context in stipe fibrous, with longitudinal stripes, shiny, yellowish brown (5B6), sometimes reddish-brown (8D6) at the base. ***Odor*** fungoid.

***Basidiospores*** [100/5/3](8.1–)8.2–8.96–9.3(–9.5) × (4.8–)4.9–5.12–5.4(–6.1) μm, Q = (1.46–)1.65–1.75–1.84(–1.88), Q_m_ ± SD = 1.75 ± 0.07, long ellipsoidal, smooth, yellowish to brownish, thick-walled, with indistinct apiculus, often containing one yellowish-green circular oil-droplet inclusion, and a few have irregular granular inclusions or two yellowish-green circular oil-droplet inclusions. ***Basidia*** 21–33 × 8.1–15 μm, short clavate to broadly clavate, some elongated clavate, apex obtuse or rounded, tapering to base, 4-spored, occasionally 2-spored, 2–5 μm long, interior with yellow or yellowish green inclusions, some colorless. ***Pleurocystidia*** absent. ***Cheilocystidia*** 29–**43.3**–58 × 7.2–**13.99**–19 μm (n = 30), clustered, abundant, clavate, with a slightly obtuse and rounded top, gradually narrowing towards the base, at the top in the balloon expands, sometimes a significant space, thin-walled, not hyaline, colorless, some containing yellow refractive pigments. ***Hymenophoral trama*** 70–120 μm thick, sub-regular arrangement, composed of colorless, transparent, regularly arranged dilated cylindrical hyphae, 4–13 μm in width. ***Pileipellis*** 60–120 μm thick, sub-regular arrangement, golden or yellowish brown when aggregated, composed of golden or yellow, inflated to cylindrical hyphae, 5–12 μm wide, encrusted. ***Pileal trama*** colorless, hyaline, made up of thin-walled and obviously inflated hyphae 6–14 μm wide. ***Stipitipellis*** hyphae 5–10 μm wide, arranged regularly, golden brown, encrusted. ***Stipe trama*** hyphae 7–11 μm wide, rule arrangement, transparent, colorless, thin-walled. ***Oleiferous*** hyphae 5–17 μm wide, present in the pileus, stipe and hymenium, yellow or yellow-green, smooth, slightly curved. ***Clamp connections*** observed in all tissues.

##### Habitat and ecology.

Gregarious or in small groups under broad-leaved forests.

##### Distribution.

China (Guangxi).

##### Additional specimens examined.

**China** • Guangxi Zhuang Autonomous Region: Hechi City, Huanjiang Maonan Autonomous County, Mulun National Nature Reserve, occurring in fagaceous forests, 22 September 2018, G.F. Mou, M2018092217 (IBK), 25 July 2020, G.F. Mou, M2020072503 (IBK), and M2020072531 (IBK); • Nanning City, Damingshan National Nature Reserve, 16 July 2022, J.R. Liu, DMS2022071619 (IBK).

#### Inosperma nonggangense

Taxon classificationFungiAgaricalesInocybaceae

Y.G. Fan, G.F. Mou & S. Li sp. nov.

7206D3F4-7A02-52EA-882B-2614B85F6CD9

Fungal Names: FN 573255

Figs 4, 5

##### Chinese name.

弄岗歧盖伞 (Pinyin: nòng gǎng qí gài sǎn).

**Figures 4.**
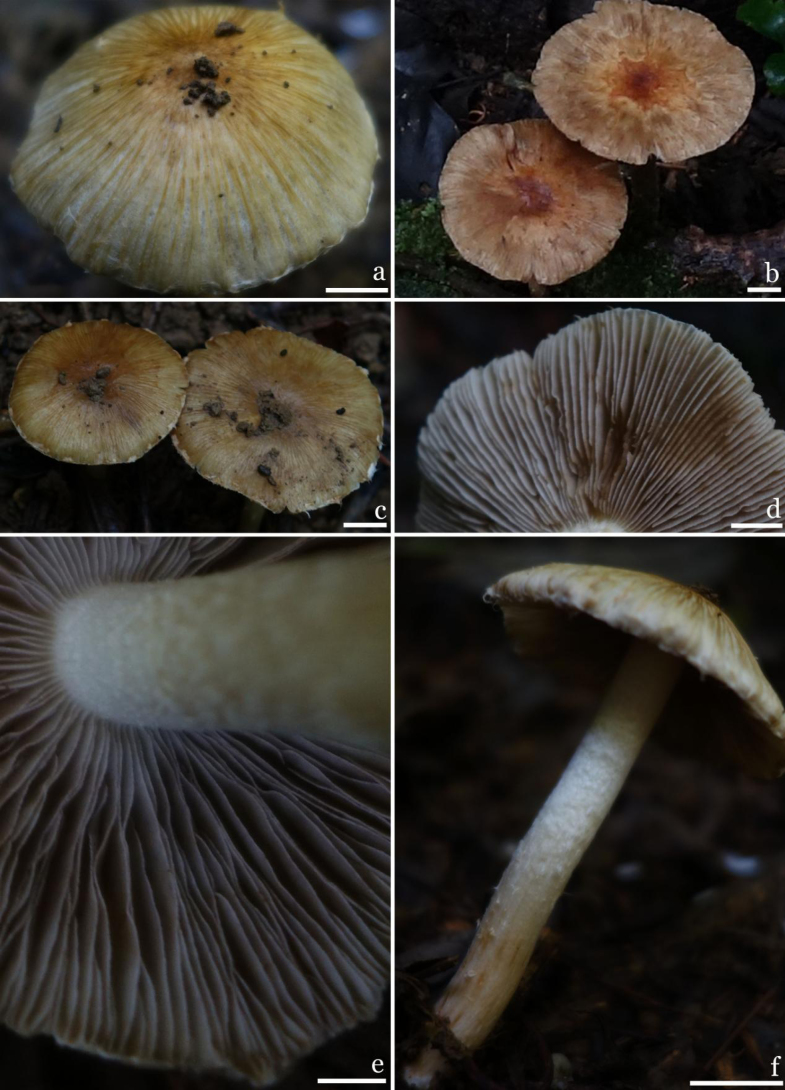
Basidiomata of *Inosperma nonggangense*. (M2018061321, holotype). **a–c**. Pileus; **d–e**. Lamellae; **f**. Stipe surface. Scale bars: 10 mm (**a–f**). Photos by G.F. Mou.

**Figures 5.**
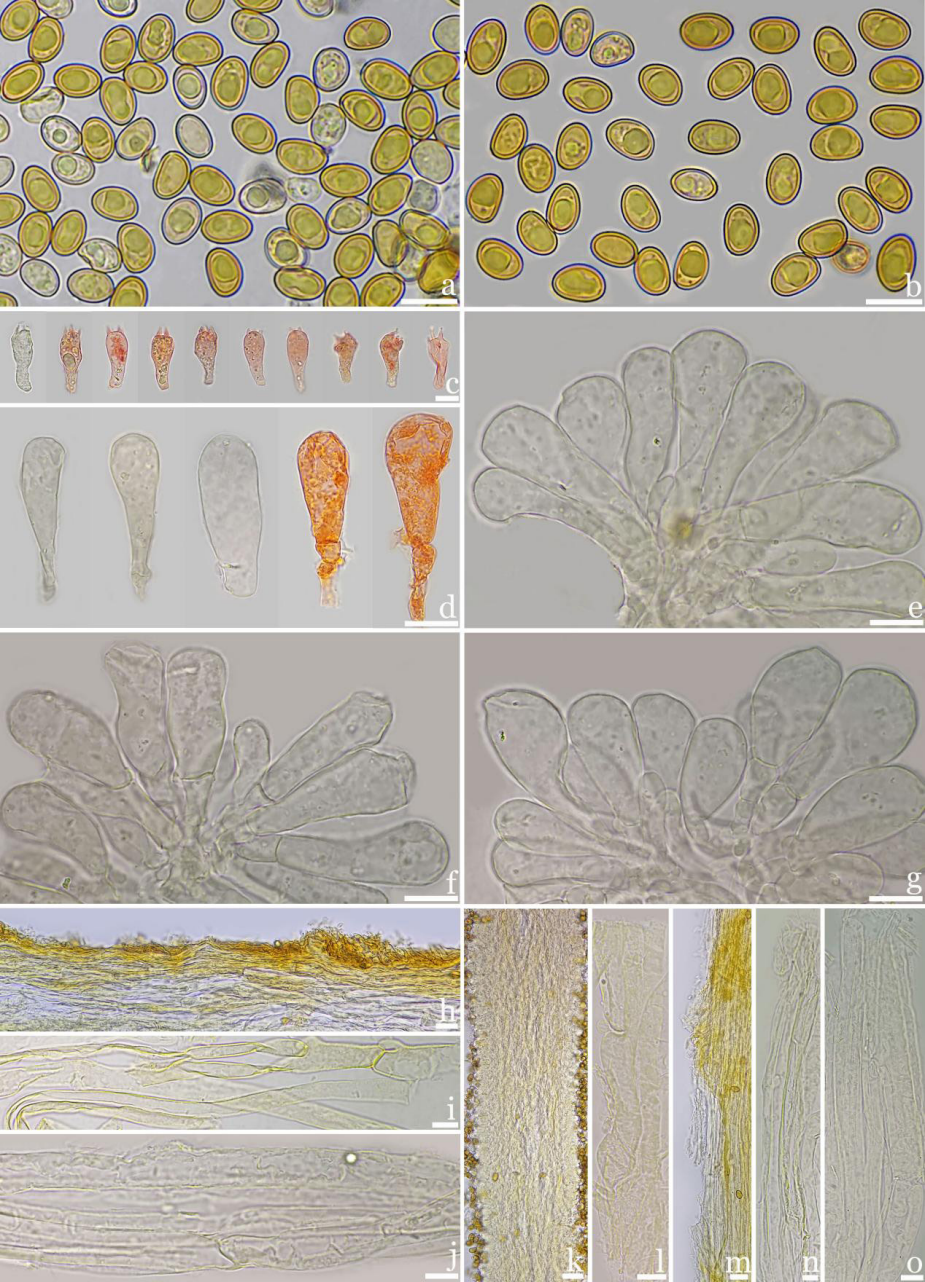
Microscopic features of *Inosperma nonggangense*. ((M2018061321, holotype). **a–b**. Basidiospores; **c**. Basidia; **d–g**. Cheilocystidia; **h**. Pileipellis; **i**. Hyphae of pileipellis; **j**. Hyphae of pileal trama; **k**. Hymenium; **l**. Hymenophoral trama; **m**. Stipitipellis; **n**. Stipitipellis hyphae; **o**. Stipe trama hyphae. Scale bars: 10 μm (**a–o**).

##### Etymology.

nonggangense (Lat.): referring to Nonggang National Nature Reserve (Guangxi Zhuang Autonomous Region, China), where the type specimen was collected.

##### Holotype.

**China** • Guangxi Zhuang Autonomous Region, Chongzuo City, Longzhou County, occurring in fagaceous forests, 22°32'11"N, 106°49'1"E, 13 June 2018, G.F. Mou, M2018061321 (IBK), GenBank accession number: (ITS: PX643276; LSU: PX629743).

##### Diagnosis.

*Inosperma nonggangense* has yellowish-brown basidiomata when mature, brownish-yellow and non-rimose pileus, crowded lamellae, stipe apex covered with pruinose particles, ovoid to broadly ellipsoid basidiospores, and thin-walled, clavate to short-clavate cheilocystidia. Most similar to *Inos. zonativeliferum*, but differs from it by the yellowish basidiomata, less veilipellis layer in pileus, lack of pinkish tinge in lamellae, and non-hyaline cheilocystidia.

##### Description.

***Basidiomata*** medium- to large-sized, clustered. ***Pileus*** 27–66 mm in diameter, conical when young, then gradually bell-shaped with a convex central, convex to spread with mature, margin of pileus slightly decurved to extended; surface dry, covered with white (1 A1) to grayish white (1 A1–1 B1) veil remnants, gradually disappear, fibrillose to indistinctly rimulose, margin cracked; brownish yellow (4A4), yellowish (4A5–4A6) to orange (6B7), center goldish (6C8) to orange red (8B7), margin yellowish (4A4). ***Lamellae*** adnexed, crowded, 3–6 mm wide, with 3–4 tiers of lamellulae; initially white (1A1), then gradually turn grayish-white (1A1–1B1) with yellowish-brown (5C7) tinges, becoming pale brown (5C5) at maturity. ***Stipe*** 32–84 × 4–10 mm, central, solid, cylindrical, slightly curved, equal; exhibiting longitudinal striate-fibrillose throughout, apices with white (1A1) powdery particles, with pure white (1A1) fibrils downwards, white (1A1) to yellowish (4B6), yellowish brown (5C6) when mature. ***Context in pileus*** white (1A1), 2–4 mm thick, up to 6 mm thick under the disc; ***context in stipe*** fibrous, with longitudinal stripes, shiny, white (1A1), basal flesh yellowish brown (4B6). ***Odor*** fungoid.

***Basidiospores*** [100/2/2] (8.2–)8.7–9.18–9.9 (–10.0) × (4.8–)5.2–5.11–6.1 (–6.1) μm, Q = (1.46–)1.47–1.75–1.81 (–1.88), Q_m_ ± SD = 1.75 ± 0.07, ovoid to broadly ellipsoid, smooth, yellowish to yellowish brown, thick-walled, with indistinct apiculus, most containing one yellow-green oil droplet, a few containing two yellow-green oil droplet, granular or no inclusions. ***Basidia*** 22–42 × 8.9–13 μm, clavate to short-clavate, apices obtuse, gradually tapered toward base, 4-spored, sometimes 2-spored, sterigmata 2–5 μm in length, transparent, mostly colorless, a few pale yellow, most with yellow-green granular or round oily inclusions. ***Pleurocystidia*** absent. ***Cheilocystidia*** 33–**45.7**–70 × 9.8–**14.9**–22 μm (n = 30), clustered, abundant, clavate to elongate-clavate, occasionally with balloon-shaped swelling at the apex, gradually tapered towards the base, some have obvious separations, thin-walled, colorless but non-hyaline, sometimes contain yellow refractive substances inside. ***Hymenophoral trama*** about 80–180 μm thick, colorless to yellowish, subregularly arranged, composed of colorless or yellow, rough, cylindrical to swollen hyphae, 5–15 μm in width. ***Pileipellis*** 27–80 μm thick, sub-regularly arranged, yellow to yellow-brown when aggregated, composed of thin-walled, encrusted, yellow, cylindrical hyphae, 6–11 μm wide. ***Pileal trama*** colorless, transparent, made up of thin-walled and obviously swollen hyphae 7–18 μm wide. ***Stipitipellis*** hyphae 7–11 μm wide, regularly arranged, yellow to yellow-brown. ***Stipe trama*** hyphae 5–14 μm wide, regularly arranged, hyaline, colorless, thin-walled. ***Oleiferous*** hyphae 4–10 μm wide, present in the pileus, stipe, and hymenohporal trama. Clamp connections observed in all tissues.

##### Habitat and ecology.

Gregarious or in small groups under broad-leaved forests.

##### Distribution.

China (Guangxi).

##### Additional specimens examined.

**China** • Guangxi Zhuang Autonomous Region: Chongzuo City, Longzhou County, Nonggang National Nature Reserve, in broad-leaved forests, 25 September 2018, G.F. Mou, M2018092505 and 26 February 2018, G.F. Mou, M2018102624 (IBK).

## Discussion

In this study, two new *Inosperma* species were discovered in Guangxi karst forests, in southern China, enriching the fungal diversity of this ecosystem. Combined morphological and multi-gene molecular analyses were employed to describe these species. The two new species, *Inos. rufobrunneum* and *Inos. nonggangense*, exhibit typical characteristics observed in tropical members of *Inosperma*. These include medium-sized basidiomata, fibrous to finely fissured or torn pileus, smooth-fibrillose stipes with sparse fibrils at the upper part, even or only slightly enlarged stipe base, elliptical spores, and absence of pleurocystidia (Pradeep et al. 2016; Latha and Manimohan 2017; Deng et al. 2021b).

In the three-gene phylogeny, *Inos. rufobrunneum* clusters sister to the lineage comprising *Inos. wuzhishanense* and *Inos. akirnum. Inosperma wuzhishanense*, recently described from tropical China, shares reddish brown pileus, crowded lamellae, and clavate thin-walled cheilocystidia, but it has a vinaceus tinge in basidiomata, appressed-fribils in pileus, more distinct fibrils on stipe surface, more ellipsoid to ovoid spores with smaller average Q values (Q_m_ = 1.50), and mostly elongate-clavate cheilocystidia that are not distinctly tapered downwards (Zhou et al. 2023). *Inosperma akirnum*, described from tropical India, has more yellowish basidiomata, smaller basidiospores measuring 7–8.5 × 5–5.5 µm, longer and narrower cheilocystidia, and an ecology among ginger plants (Latha and Manimohan 2017). In addition, the Indian species *Inos. saragum* (K.P.D. Latha & Manim.) Matheny & Esteve-Rav. is similar to *Inos. rufobrunneum* in having ellipsoid spores and clavate cheilocystidia, but the former has more yellowish basidiomata, appressed- to slightly recurved squamulose around the disc, clavate to utriform cheilocystidia with a median constriction, and an ecology near *Hopea ponga* (Dipterocarpaceae) (Latha and Manimohan 2017).

*Inosperma nonggangense* is phylogenetically related to several species in the Old World tropical clade 2. These include *Inos. gregarium* (K.P.D. Latha & Manim.) Matheny & Esteve-Rav., *Inos. hainanense* Y.G. Fan, L.S. Deng, W.J. Yu & N.K. Zeng, *Inos. muscarium* Y.G. Fan, L.S. Deng, W.J. Yu & N.K. Zeng, *Inos. virosum* (K.B. Vrinda, C.K. Pradeep, A.V. Joseph & T.K. Abraham ex C.K. Pradeep, K.B. Vrinda & Matheny) Matheny & Esteve-Rav., and *Inos. zonativeliferum* Y.G. Fan, H.J. Li, F. Xu, L.S. Deng & W.J. Yu. However, the two tropical Indian species, *Inos. gregarium* and *Inos. virosum* both have brownish basidiomata, smaller basidiospores (*Inos. gregarium*: 7–8.5 × 5–5.5 μm; *Inos. virosum*: 6.5–8.5 × 5–6 μm), and transparent cheilocystidia, (Latha and Manimohan 2017). *Inosperma hainanense* and *Inos. muscarium*, recently described from China, have brownish basidiomata, a rimulose to rimose pileus, as well as transparent cheilocystidia (Deng et al. 2021b). *Inosperma zonativeliferum* occasionally shares a similar pileus color with *Inos. nonggangense*, but it has a heavy layer of grayish zonate velipellis on the pileus, relatively wider spores with an average Q value of 1.57, and broader and more transparent cheilocystidia measuring 30–58 × 16–24 μm. (Deng et al. 2022).

Muscarine is the principal toxin found in *Inosperma*. It binds to acetylcholine receptors and induces characteristic activation of the parasympathetic nervous system. The classic symptom triad consists of sweating, salivation, and lacrimation (Lurie et al. 2009; White et al. 2018). *Inosperma erubescens* (A. Blytt) Matheny & Esteve-Rav. was responsible for mass human poisonings in Germany as early as 1963 (Herrmann 1964; Lurie et al. 2009). Within Old World tropical clade 2, Parnmen et al. (2021) documented ten cases of poisoning by *Inosperma* species in Thailand between 2010 and 2018. *Inosperma muscarium*, *Inos. hainanense*, *Inos. zonativeliferum*, and several undescribed taxa have also been implicated in multiple poisoning incidents in tropical China (Li et al. 2021, 2022; Deng et al. 2022). Further investigation is warranted regarding the toxin profiles of the two new species described in this study.

## Supplementary Material

XML Treatment for Inosperma rufobrunneum

XML Treatment for Inosperma nonggangense

## Additional information

### Artificial Intelligence (AI) use

The authors accept full responsibility for the content of the manuscript, including the disclosure of any use of AI.

No AI tools were used in the preparation of this manuscript.

### Author ORCIDs

Y.C. Zhang https://orcid.org/0009-0000-5278-1134

S. Li https://orcid.org/0009-0005-5325-8923

Y-G. Fan https://orcid.org/0000-0002-3028-9752

J.R. Liu https://orcid.org/0000-0003-0843-2097

W.N. Tan https://orcid.org/0000-0002-5274-4429

Y. Liu https://orcid.org/0000-0001-8931-9656

G.F. Mou https://orcid.org/0009-0007-2318-7807

### Data availability

All of the data that support the findings of this study are available in the main text or Supplementary Information.

## Supplementary materials

Supplementary material 1 Supplementary table SS1 Data type xlsx Explanation note Taxa, sequences and collections analyzed in this study. Collection in bold are newly sequenced in this study. https://binary.pensoft.net/file/1657896This dataset is made available under the Open Database License (http://opendatacommons.org/licenses/odbl/1.0/). The Open Database License (ODbL) is a license agreement intended to allow users to freely share, modify, and use this Dataset while maintaining this same freedom for others, provided that the original source and author(s) are credited. Yan Cheng Zhang, Shuang Li, Yu-Guang Fan, Jin Rong Liu, Wei Ning Tan, Yan Liu, Guang Fu Mou

## References

[B1] Aïgnon HL, Jabeen S, Naseer A, Yorou NS, Ryberg M (2021) Three new species of *Inosperma* (Agaricales, Inocybaceae) from tropical Africa. MycoKeys 77: 97–116. 10.3897/mycokeys.77.60084 PMC7862217 33551659

[B2] Aïgnon HL, Fan YG, De Kesel A, Bahram M, Ryberg M, Yorou NS (2023) A new species of *Inosperma*, and first record of *I. afromelliolens* (Inocybaceae, Fungi) from West Africa. PLOS ONE 18(10): e0290894. 10.1371/journal.pone.0290894 PMC10584187 37851619

[B3] Bandini D, Oertel B, Eberhardt U (2021) Even more fibre-caps (2): Thirteen new species of the family Inocybaceae. Mycologia Bavarica 21: 27–98.

[B4] Bandini D, Oertel B, Eberhardt U (2022) Even more fibre-caps (3): Twenty-one new species of the family Inocybaceae. Mycologia Bavarica 22: 31–138. 10.18476/2022.901982

[B5] Bao DF, Tian XG, Samarakoon MC, Karunarathna SC, Luo ZL, Han JJ, Wen TC, He ZJ, Liu ZH, Lu YZ, Kang JC (2025) Biodiversity of lignicolous freshwater fungi from the Nanpan river basin in Guizhou and Guangxi provinces, China, with descriptions of fifteen species. Mycosphere 16(1): 3695–3752. 10.5943/mycosphere/16/1/29

[B6] Bau T, Fan YG (2018) Three new species of *Inocybe* sect. *Rimosae* from China. Mycosystema 37(6): 693–702.

[B7] Bizio E, Castellan A (2017) *Inocybe acutofulva* e *Inocybe grammopodia* var. *paleoveneta*, duenuovi taxa dall’alta marca trevigiana (Treviso, Veneto, Italia). Micologia Vegetazione Mediterranea 32(2): 103–124.

[B8] Cervini M, Carbone M, Bizio E (2020) *Inosperma vinaceum*, una nuova specie distinta da *I. rhodiolum* e *I. adaequatum*. Rivista di Micologia 63(3): 215–241.

[B9] Chandrasekharan B, Pradeep C, Vrinda B (2020) *Inocybe* poisoning from Kerala—a case study. Journal of Mycological Research 57(4): 255–258.

[B10] Chen HL, Huang YS (2025) General report on new taxa of vascular plants in Guangxi. Guihaia 45(3): 396–405. 10.11931/guihaia.gxzw202502033

[B11] Crous P, Jurjevi Z, Balashov S, Pinruan AM, Rigueiro-Rodriguez A, Osieck ER, Altes A, Czachura P, Esteve-Raventos F, Gunaseelan S, Kaliyaperumal M, Larsson E, Luangsa-Ard JJ, Moreno G, Pancorbo F, Pitek M, Sommai S, Somrithipol S, Asif M, Delgado G, Flakus A, Illescas T, Kezo K, Khamsuntorn P, Kubatova A, Labuda R, Lavoise C, Lebel T, Lueangjaroenkit P, Macia-Vicente J, Paz A, Saba M, Shivas R, Tan Y, Wingfield M, Aas T, Abramczyk B, Ainsworth A, Akulov A, Alvarado P, Armada F, Assyov B, Avchar R, Avesani M, Bezerra J, Bhat J, Bilaski P, Bily D, Boccardo F, Bozok F, Campos J, Chaimongkol S, Chellappan N, Costa M, Dalecka M, Darmostuk V, Daskalopoulos V, Dearnaley J, Dentinger B, Silva ND, Dhotre D, Carlavilla J, Doungsa-ard C, Dovana F, Erhard A, Ferro L, Gallegos S, Giles C, Gore G, Gorfer M, Guard F, Hanson S, Haridev P, Jankowiak R, Jeffers S, Kandemir H, Karich A, Kiso K, Kiss L, Krisai-Greilhuber I, Latha K, Lorenzini M, Lumyong S, Manimohan P, Manjon J, Maula F, Mazur E, Mesquita N, Mynek K, Mongkolsamrit S, Moran P, Murugadoss R, Nagarajan M, Nalumpang S, Noisripoom W, Nosalj S, Novaes Q, Nowak M, Pawowska J, Peiger M, Pereira O, Pinto A, Plaza M, Polemis E, Polhorsky A, Ramos D, Raza M, Rivas-Ferreiro M, Rodriguez-Flakus P, Ruszkiewicz-Michalska M, Sanchez A, Santos A, Schuller A, Scott P, Shelke D, Liwa L, Solheim H, Sonawane H, Straiftakova D, Stryjak-Bogacka M, Sudsanguan M, Suwannarach N, Suz L, Syme K, Takn H, Tennakoon D, Tomka P, Vaghefi N, Vasan V, Vauras J, Wiktorowicz D, Villarreal M, Vizzini A, Wrzosek M, Yang X, Yingkunchao W, Zapparoli G, Zervakis G, Groenewald J (2024) Fungal Planet description sheets: 1614–1696. Fungal Systematics and Evolution 13: 183–440. 10.3114/fuse.2024.13.11 PMC11320056 39140100

[B12] Deng LS, Yu WJ, Zeng NK, Liu LJ, Liu LY, Fan YG (2021a) *Inosperma subsphaerosporum* (Inocybaceae), a new species from Hainan, tropical China. Phytotaxa 502: 169–178. 10.11646/phytotaxa.502.2.5

[B13] Deng LS, Kang R, Zeng NK, Yu WJ, Chang C, Xu F, Deng WQ, Qi LL, Zhou YL, Fan YG (2021b) Two new *Inosperma* (Inocybaceae) species with unexpected muscarine contents from tropical China. MycoKeys 85: 87–108. 10.3897/mycokeys.85.71957 PMC8695569 35035255

[B14] Deng LS, Yu WJ, Zeng NK, Zhang YZ, Wu XP, Li HJ, Xu F, Fan YG (2022) A new muscarine-containing *Inosperma* (Inocybaceae, Agaricales) species discovered from one poisoning incident occurring in tropical China. Frontiers in Microbiology 13: 923435. 10.3389/fmicb.2022.923435 PMC9290438 35859745

[B15] Esteve-Raventós F, Larsson E, Pancorbo F, Bizio E, Altés A, Turégano Y, Moreno G, Olariaga I (2024) A taxonomic and phylogenetic contribution on *Inosperma* section *Inosperma* (Agaricales, Inocybaceae) in Europe: Calamistratum and Geraniodorum Groups. Journal of Fungi 10(6): 374. 10.3390/jof10060374 PMC11205153 38921361

[B16] Gardes M, Bruns TD (1993) ITS primers with enhanced specificity for basidiomycetes– application to the identification of mycorrhizae and rusts. Molecular Ecology 2(2): 113–118. 10.1111/j.1365-294X.1993.tb00005.x 8180733

[B17] Herrmann M (1964) Die Naumburger Massen-Pilzvergiftung mit dem Ziegelroten Rißpilz–*Inocybe patouillardii*. Mykologisches Mitteilungsblatt 8: 42–44.

[B18] Horak E, Matheny PB, Desjardin DE, Soytong K (2015) The genus *Inocybe* (Inocybaceae, Agaricales, Basidiomycota) in Thailand and Malaysia. Phytotaxa 230(3): 201–238. 10.11646/phytotaxa.230.3.1

[B19] Hall TA (1999) BioEdit: A user-friendly biological sequence alignment editor and analysis program for Windows 95/98/NT. Nucleic Acids Symposium Series 41(41): 95–98.

[B20] Kuyper TW (1986) A revision of the genus *Inocybe* in Europe. I. Subgenus *Inosperma* and the smooth-spored species of subgenus *Inocybe*. Persoonia Supplement 3: 1–247.

[B21] Kornerup A, Wanscher JH, Pavey D (1978) Methuen handbook of colour. Methuen Publishing Ltd, London, 256 pp.

[B22] Kühner R (1980) Les Hymenomycetes agaricoides. Bulletin Mensuel de la Societe Linneenne de Lyon 49: 11027.

[B23] Kropp BR, Matheny PB, Hutchison LJ (2013) *Inocybe* section *Rimosae* in Utah: phylogenetic affinities and new species. Mycologia 105(3): 728–747. 10.3852/12-185 23233513

[B24] Kumar S, Stecher G, Tamura K (2016) MEGA7: molecular evolutionary genetics analysis version 7.0 for bigger datasets. Molecular Biology and Evolution 33(7): 1870–1874. 10.1093/molbev/msw054 PMC8210823 27004904

[B25] Lurie Y, Wasser SP, Taha M, Shehade H, Nijim J, Hoffmann Y, Basis F, Moshe V, Lavon O, Suaed S, Bishara B, Bentur Y (2009) Mushroom poisoning from species of genus *Inocybe* (fiber head mushroom): A case series with exact species identification. Clinical Toxicology 47: 562–565. 10.1080/15563650903008448 19566380

[B26] Latha KPD, Manimohan P (2016) *Inocybe gregaria*, a new species of the *Inosperma* clade from tropical India. Phytotaxa 286(2): 107–115. 10.11646/phytotaxa.286.2.5

[B27] Latha KPD, Manimohan P (2017) Inocybes of Kerala. SporePrint Books, Calicut-India, 181 pp.

[B28] Li HJ, Zhang HS, Zhang YZ, Zhou J, Yin Y, He Q, Jiang SF, Ma PB, Zhang YT, Wen K, Yuan Y, Lang N, Cheng BW, Lu JJ, Sun CY (2021) Mushroom poisoning outbreaks-China, 2020. China CDC Weekly 3(3): 41–45. 10.46234/ccdcw2021.014 PMC8392932 34594953

[B29] Li S, Xu F, Long P, Zhang P, Fan YG, Chen ZH (2022) Five new species of *Inosperma* from China: Morphological characteristics, phylogenetic analyses, and toxin detection. Microbiology 13: 1021583. 10.3389/fmicb.2022.1021583 PMC9659589 36386664

[B30] Liu LL, Ren YL, Habib K, Lu CT, Wu YP, Long SH, Lin Y, Zhang X, Kang YQ, Wijayawardene NN, Wang F, Elgorban AM, Rajaei AI, Samarakoon MC, Shen XC, Li QR (2025) New taxa of Xylariales from Karst Ecosystems in Southwestern China. Mycosphere 16(1): 1–78. 10.5943/mycosphere/16/1/1

[B31] Matheny PB (2005) Improving phylogenetic inference of mushrooms with *RPB1* and *RPB2* nucleotide sequences (*Inocybe*, Agaricales). Molecular Phylogenetics and Evolution 35(1): 1–20. 10.1016/j.ympev.2004.11.014 15737578

[B32] Matheny PB, Curtis JM, Hofstetter V, Aime MC, Moncalvo JM, Ge ZW, Slot JC, Ammirati JF, Baroni TJ, Bougher NL, Hughes KW, Lodge DJ, Kerrigan RW, Seidl MT, Aanen DK, DeNitis M, Daniele GM, Desjardin DE, Kropp BR, Norvell LL, Hibbett DS (2006) Major clades of Agaricales: a multilocus phylogenetic overview. Mycologia 98(6): 982–995. 10.1080/15572536.2006.11832627 17486974

[B33] Matheny PB, Moreau P-A (2009a) A rare and unusual lignicolous species of *Inocybe* (Agaricales) from eastern North America. Brittonia 61: 163–171. 10.1007/s12228-008-9066-4

[B34] Matheny PB, Aime MC, Bougher NL, Buyck B, Desjardin DE, Horak E, Kropp BR, Lodge DJ, Soytong K, Trappe JM, Hibbett DS (2009b) Out of the Palaeotropics? Historical biogeography and diversification of the cosmopolitan ectomycorrhizal mushroom family Inocybaceae. Journal of Biogeography 36(4): 577–592. http://www.jstor.org/stable/20488391

[B35] Marchetti M, Franchi P, Consiglio G (2014) Typification of some of Britzelmayr’s *Inocybe* species. Revista Iberoamericana De Micología 2014(2): 127–178.

[B36] Matheny PB, Henkel TW, Séné O, Korotkin HB, Dentinger BTM, Aime MC (2017) New species of *Auritella* (Inocybaceae) from Cameroon, with a worldwide key to the known species. IMA Fungus 8: 287–298. 10.5598/imafungus.2017.08.02.06 PMC5729713 29242776

[B37] Matheny PB, Hobbs AM, Esteve-Raventos F (2020) Genera of Inocybaceae: New skin for the old ceremony. Mycologia 112(1): 83–120. 10.1080/00275514.2019.1668906 31846596

[B38] Naseer A (2018) *Inocybe shawarensis* sp. nov. in the *Inosperma* clade from Pakistan. Mycotaxon 132(4): 909–918. 10.5248/132.909

[B39] Naseer A, Khurshid R, Fan YG, Khalid AN (2025) Morphology and multigene phylogeny reveal a new species of *Inosperma* from Himalayas of Pakistan. BMC Microbiology 25: 688. 10.1186/s12866-025-03972-y PMC12557976 41146017

[B40] Nylander J (2004) MrModeltest V2. program distributed by the author. Bioinformatics 24: 581–583.

[B41] Pradeep CK, Vrinda KB, Varghese SP, Korotkin HB, Matheny PB (2016) New and noteworthy species of *Inocybe* (Agaricales) from tropical India. Mycological Progress 15: 24. 10.1007/s11557-016-1174-z

[B42] Parnmen S, Nooron N, Leudang S, Sikaphan S, Polputpisatkul D, Pringsulaka O, Binchai S, Rangsiruji A (2021) Foodborne illness caused by muscarine-containing mushrooms and identification of mushroom remnants using phylogenetics and LC–MS/MS. Food Control 128(4): 108182. 10.1016/j.foodcont.2021.108182

[B43] Rathnayaka AR, Tennakoon DS, Jones GE, Wanasinghe DN, Bhat DJ, Priyashantha AH, Stephenson SL, Tibpromma S, Karunarathna SC (2025) Significance of precise documentation of hosts and geospatial data of fungal collections, with an emphasis on plant-associated fungi. New Zealand Journal of Botany 63(2–3): 462–489. 10.1080/0028825X.2024.2381734

[B44] Ronquist F, Teslenko M, van der Mark P, Ayres DL, Darling A, Höhna S, Larget B, Liu L, Suchard MA, Huelsenbeck JP (2012) MrBayes 3.2: efficient Bayesian phylogenetic inference and model choice across a large model space. Systematic Biology 61(3): 539–542. 10.1093/sysbio/sys029 PMC3329765 22357727

[B45] Sana S, Kiran M, Ali W (2025) *Inosperma pakistanicum* sp. nov. (Inocybaceae, Agaricales) from Swat, Pakistan. Phytotaxa 690(1): 77–88. 10.11646/phytotaxa.690.1.6

[B46] Tan WN, Luo LJ, Nong SY, Hang XQ, Liu Y, Huang YS (2023) Primary study on species diversity of plant in Guangxi Mulun national nature reserve. Guihaia 43(12): 2182–2195.

[B47] Trifinopoulos J, Nguyen LT, von Haeseler A, Minh BQ (2016) W-IQ-TREE: a fast online phylogenetic tool for maximum likelihood analysis. Nucleic Acids Research 44(W1): 1–4. 10.1093/nar/gkw256 PMC4987875 27084950

[B48] Vilgalys R, Hester M (1990) Rapid genetic identification and mapping of enzymatically amplified ribosomal DNA from several *Cryptococcus* species. Journal of Bacteriology 172(8): 4238–4246. 10.1128/jb.172.8.4238-4246.1990 PMC213247 2376561

[B49] Wu XL, Tan WF, Song B, Peng DR, Wu WH (2021) Guangxi macrofungus in China. China Forestry Publishing House, Beijing, 447 pp.

[B50] White J, Weinstein S, Haro LD, Bédry R, Schaper A, Rumack BH, Zilker T (2018) Mushroom poisoning: A proposed new clinical classification. Toxicon 157: 53–65. 10.1016/j.toxicon.2018.11.007 30439442

[B51] Zhou YL, Deng LS, Yang SD, Liu CF, Fan YG, Yu WJ (2023) Phylogenetic analysis, morphological studies, element profiling, and muscarine detection reveal a new toxic *Inosperma* (Inocybaceae, Agaricales) species from tropical China. Microbiology 14: 1326253. 10.3389/fmicb.2023.1326253 PMC10740167 38143868

